# Reliance on Visual Input for Balance Skill Transfer in Older Adults: EEG Connectome Analysis Using Minimal Spanning Tree

**DOI:** 10.3389/fnagi.2021.632553

**Published:** 2021-02-04

**Authors:** Yi-Ching Chen, Yu-Chen Chou, Ing-Shiou Hwang

**Affiliations:** ^1^Department of Physical Therapy, College of Medical Science and Technology, Chung Shan Medical University, Taichung City, Taiwan; ^2^Physical Therapy Room, Chung Shan Medical University Hospital, Taichung City, Taiwan; ^3^Department of Physical Therapy, College of Medicine, National Cheng Kung University, Tainan City, Taiwan; ^4^Institute of Allied Health Sciences, College of Medicine, National Cheng Kung University, Tainan City, Taiwan

**Keywords:** EEG, balance, graph analysis, human aging, generalization

## Abstract

Skill transfer from trained balance exercises is critical to reduce the rate of falls in older adults, who rely more on vision to control postural responses due to age-dependent sensory reweighting. With an electroencephalography (EEG) minimum spanning tree (MST) structure, the purpose of this study was to compare the organization of supraspinal neural networks of transfer effect after postural training using full and intermittent visual feedbacks for older adults. Thirty-two older adults were randomly assigned to the stroboscopic vision (SV) (*n* = 16; age = 64.7 ± 3.0 years) and control (16; 66.3 ± 2.7 years) groups for balance training on a stabilometer (target task) with on-line visual feedback. Center-of-pressure characteristics and an MST-based connectome of the weighted phase-lag index during the bilateral stance on a foam surface (transfer task) were compared before and after stabilometer training. The results showed that both the SV and control groups showed improvements in postural stability in the trained task (*p* < 0.001). However, unlike the control group (*p* = 0.030), the SV group who received intermittent visual feedback during the stabilometer training failed to reduce the size of postural sway in the anteroposterior direction of the postural transfer task (unstable stance on the foam surface) in the post-test (*p* = 0.694). In addition, network integration for the transfer task in the post-test was absent in the SV group (*p* > 0.05). For the control group in the post-test, it manifested with training-related increases in leaf fraction in beta band (*p* = 0.015) and maximum betweenness in alpha band (*p* = 0.018), but a smaller diameter in alpha (*p* = 0.006)/beta (*p* = 0.021) bands and average eccentricity in alpha band (*p* = 0.028). In conclusion, stabilometer training with stroboscopic vision impairs generalization of postural skill to unstable stance for older adults. Adequate visual information is a key mediating factor of supraspinal neural networks to carry over balance skill in older adults.

## Introduction

The integration of the somatosensory, visual and vestibular systems produces suitable orientation to maintain postural stability. As aging is associated with progressive declines in the vestibular, visual, and somatosensory systems, people 60 years or older are at greater risk for injurious falls, as they become increasingly difficult to interpret the sensory information to environmental changes (Woollacott et al., 1986). Age-dependent sensory ambiguity can be minimized by down-weighting the most affected channel, and each sensory channel can mutually compensate for fractional impairment of another sensory system (Hay et al., 1996; Oie et al., 2002; Jeka et al., 2010). For sizable proprioceptive degeneration (Lord et al., 1994), older adults are inclined to adhere more strongly to visual input for postural control (Sundermier et al., 1996; McChesney and Woollacott, 2000). Various interventions with visual context have been developed to improve stance control. The common use of an unstable board (such as a stabilometer) with biofeedback allows individuals to visualize their body asymmetry and facilitates essential postural adjustments against destabilization (Chiviacowsky et al., 2010). In general, trained older subjects can improve their performance in stabilometer balance tests (Chiviacowsky et al., 2010; Allahverdipour et al., 2020). Recently, stroboscopic vision (or intermittent vision) has been proposed to improve stance control (Assländer et al., 2013; Kim et al., 2020). Continuous visual inflow is aliased by lenses of stroboscopic eyewear, which provides intermittent visual samples by alternating between clear and opaque states at a pre-set duty cycle and frequency. Stroboscopic exposure alters the capacity of perceptual or cognitive abilities to perform a motor skill, in relation to increases in visual attention for anticipatory timing (Smith and Mitroff, 2012) and motion detection (Appelbaum et al., 2012). For young athletes, stroboscopic visual training on many sport activities appears to be promising (Fransen et al., 2017; Hülsdünker et al., 2019), although it has not been integrated into balance training for older adults.

Positive motor transfer is defined as the capacity to successfully apply what is learned in one context to other contexts beyond one's limited experiences (Poggio and Bizzi, 2004). The cognitive, motor and visual networks interact, linking various motor memories into overlapping representations to generalize the motor program. The ability of motor transfer is affected by attention focus and feedback stimuli, because relevant contextual cues determine what architecture of the brain networks is used (Poggio and Bizzi, 2004) and how motor memory is encoded in the history of learning (Krakauer et al., 2006; Herszage and Censor, 2018). For instance, although subjects learn to adapt visuomotor rotation in the trained direction, the newly acquired motor skill is less transferrable to the untrained direction when attention is divided (Bédard and Song, 2013; Wang and Song, 2017). Generalization is hypothesized to take place in the initial phase of learning, when false motor traces and irrelevant noises are incorporated to form novel motor memory. As predicted by the over-fitted model of the stimulus (Sagi, 2011; Censor, 2013), extended practice specializes a motor skill by shaping the false traces in the late learning phase and reducing abstract representation and goal-based generalization.

The generalizability of motor training results to older adults is an issue of debate. The inability of older adults to adapt a trained motor skill to a novel situation has been reported (Sosnoff and Newell, 2006; Walter et al., 2019), despite such an age effect on motor transfer appearing to be incoherent across studies (Seidler, 2007; König et al., 2019). Besides, older adults have reduced interlimb transfer of the upper limbs (Barnhoorn et al., 2016) and lower limbs (Krishnan et al., 2018) in comparison with young adults. Potential generalization deficits for the elderly are multi-factorial, attributable to declines in hippocampal and fronto-striatal function to emphasize relevant stimuli (Krishna et al., 2012) and/or the limited availability of the attentional network to process visuospatial information (Bédard and Song, 2013; Lingo VanGilder et al., 2020). Hence, stroboscopic vision might be beneficial to generalize postural training to sensory environments with a higher probability of falls.

To date, little research has examined the transfer effect of trained postural skills with visual context in older adults, whose posture regulation has a higher dependence on the visual field. Considering the fact that transfer effect of motor skill involves broadly with attentional network and other neural circuits (such as fronto-parietal and fronto-temporal cortical circuits) (Obayashi, 2004), this study used minimal spanning tree (MST) to characterize coordinated interplay within inter-regional cortical activities. MST is a new perspective to highlight the core properties of information flow within EEG connectome by including the high-probability connections of all the shortest paths without loops in the network (Stam et al., 2014; van Diessen et al., 2015). The MST method has high sensitivity to small network differences (van Diessen et al., 2015) and can minimize the problems of large number of noisy connections and arbitrary threshold selection for weighted networks (Tewarie et al., 2014). With the MST approach, it was hypothesized that, when the visual feedback of older adults is intermittently occluded, the effect of stabilometer balance training would be more transferable to unstable bilateral stance on a foam surface due to the superior integration of brain networks for the postural transfer task. Reorganization of supraspinal neural network supports that transfer effect of balance training is visually-dependent, a translational observation to guide the use of exercise for falls prevention.

## Methods

### Subjects

This study recruited thirty-two recreationally active elderly participants over 60 years old (age: 65.5 ± 3.0, 15 males, 17 females) from the local community. They had normal or corrected-to-normal vision and no known cognitive problems, history of falls, or diagnoses of neurological/ musculoskeletal/metabolic disorders requiring medication. The participants were randomly assigned to the stroboscopic vision (SV) (*n* = 16; eight males, eight females; age: 64.7 ± 3.0 years) and control (*n* = 16; seven males, nine females; age: 66.3 ± 2.7 years) groups to train postural stability on the stabilometer under two visual conditions. This study was approved by an authorized institutional human research review board at the University Hospital (A-ER-107-099-T). All subjects signed the consent form before the experiment, in accordance with the Declaration of Helsinki.

### Experimental Procedures

**Figure 1:**
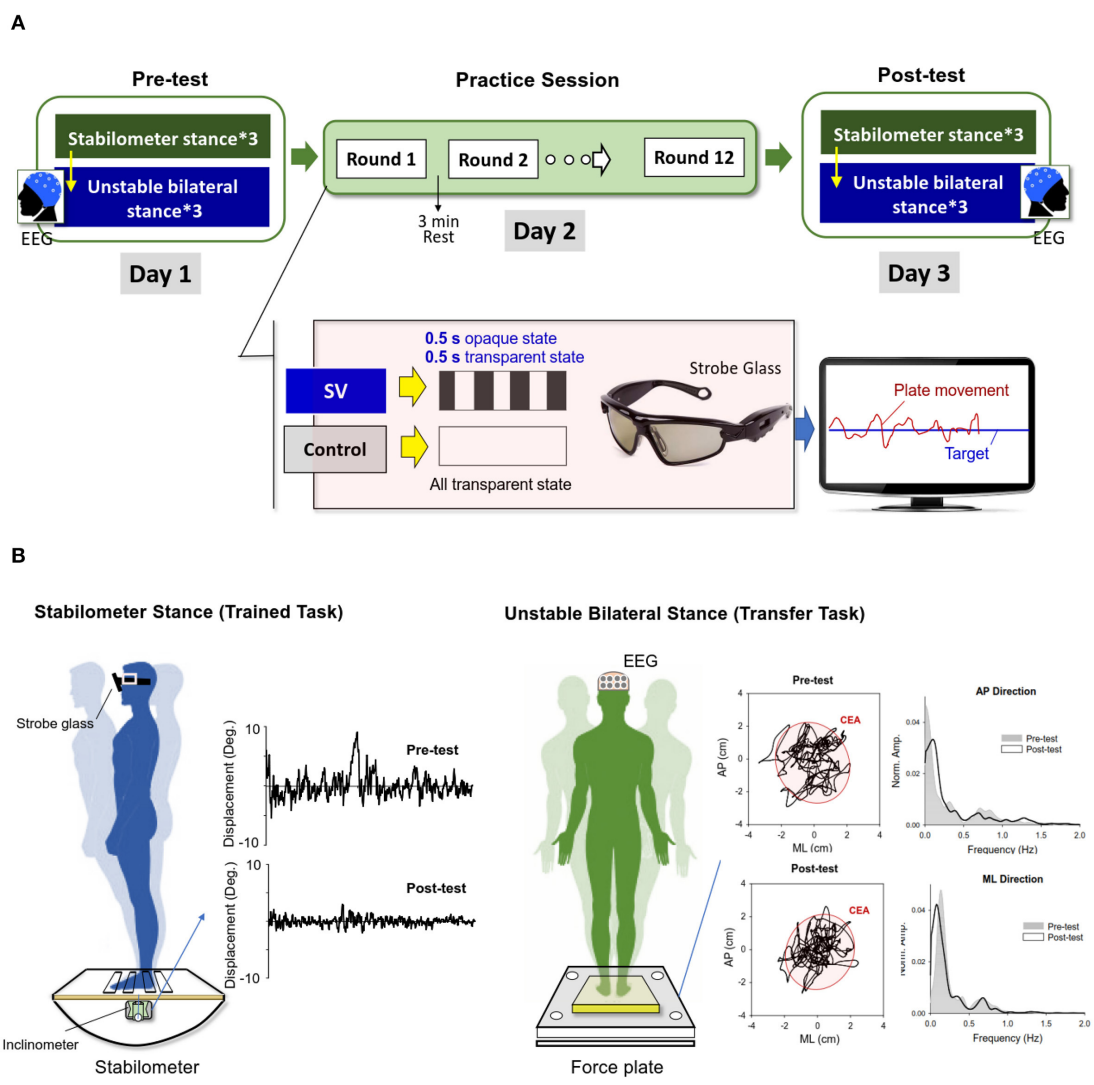
**(A)** Experimental training protocol. The pre-test (Day1) and post-test (Day 3) consisted of the trained stabilometer task and the transfer task of unstable bilateral stance. During the practice session (Day 2), the stroboscopic vision (SV) group were trained to master the stabilometer stance with intermittent visual feedback of 1 Hz, in comparison to the control group, who were trained with full vision. With online visual feedback, the participants coupled the positional trace of the stabilometer plate to the target line, which represented the level surface during the practice sessions. **(B)** Typical postural sway measures of angular displacements of the stabilometer plate during the stabilometer stance and trajectories of the center of pressure (COP) during the unstable bilateral stance in the pre-test and post-test. The power spectra of the COP trajectory in the anteroposterior (AP) and medio-lateral (ML) directions before and after practice are compared (CEA: 95% confidence ellipse sway area).

This study used a randomized, repeated measures, between-groups, parallel design. The participants were requested to visit the laboratory on three consecutive days (Figure 1A). At the first visit (Day 1), the demographic data of the participants were gathered before the pre-test measures. The pre-test measures were used to determine each participant's baseline performance of stabilometer stance without vision occlusion (training task) and unstable bilateral stance on a foam surface (transfer task) before the postural training on Day 2. The participants started by completing three trials of stabilometer stance of 45 s, interleaved with 3-min rest periods. The stabilometer task required the participants to wear a pair of stroboscopic glasses that were not in the opaque state and maintain a bilateral upright stance on a stabilometer (a wooden platform: 67 × 50 cm) with a consistent curved base (radius: 24 cm) with the arms hanging by their sides (Figure 1B). A monitor that displayed the angular movement of the stabilometer plate and the horizontal target line was placed 70 cm in front of the subject at eye level. With the visual feedback, the participants were able to maintain a steady stance on the stabilometer by carefully coupling the plate movement to the target line, which represented the ankle neutral position. After ample rest of at least 5 min, the baseline transfer task commenced. In this task, the participants maintained a bilateral upright stance as steadily as possible on an unstable foam surface (Airex Balance-pad, Switzerland) for 60 s with the eyes open (Figure 1B). There were three trials of the transfer task with 3 min of rest between trials for each participant.

At the second visit (Day 2), the participants were trained with the balance task on the stabilometer under one of two conditions: stroboscopic vision (the SV group) or full vision (the control group) (Figure 1A). Each participant wore a pair of stroboscopic glasses (Visionup Athlete VA11-AF, Japan) during the practice session. For the SV group, the stroboscopic glasses provided a 0.5-s opaque state and a 0.5-s transparent state at a rate of 1 Hz to reduce the amount of visual input from the feedback by half. They were encouraged to minimize plate movement fluctuations with intermittent visual feedback during the training rounds. The participants in the control group wore the same eyewear, but without the opaque state, so that the visual feedback to guide the posture balance on the stabilometer was not affected. There were 12 training rounds of 45 s on the stabilometer during the training session. Resting periods of 3 min were scheduled between successive training rounds. During each training round, they learned to couple the positional trace of the angular plate movement to the target line. The SV group completed the training rounds with reduced information from visual feedback, whereas the control group was trained with full vision, without any visual blocking.

At the third visit (Day 3), the participants repeated the stabilometer task and foam standing on the first visit (Day 1).

### Instrumentation Setting

A 40-channel NuAmps amplifier (NeuroScan Inc., EI Paso, USA) with Ag-AgCl scalp electrodes was used to record scalp voltage fluctuations from different cortical areas (Fp_1/2_, F_z_, F_3/4_, F_7/8_, FT_7/8_, FC_z_, FC_3/4_, C_z_, C_3/4_, CP_z_, CP_3/4_, P_z_, P_3/4_, T_3/4_, T_5/6_, TP_7/8_, O_z_, and O_1/2_). The ground electrode was placed along the midline ahead of F_z_. For off-line horizontal electrooculography (EOG) assessment, two electrodes were placed at the outer canthus of the left and right eye, respectively. For off-line vertical EOG assessment, two electrodes were placed infra- and supra-orbitally at the right eye, respectively. The impedances of all the electrodes were below 5 kΩ and were referenced to linked mastoids of both sides. The plate movements were digitized to 1 kHz with an analog-to-digital converter (Model 6341, National Instruments, USA), controlled by a Labview program (Labview v8.5, National Instruments, USA). The EEG data were recorded with a band-pass filter set at 0.1–100 Hz and a sampling rate of 1 kHz. Angular movements of the stabilometer plate were measured with an inclinometer (Model FAS-A, LORD MicroStrain, USA) mounted on the center of the stabilometer. Synchronized with EEG, the angular displacements of the stabilometer plate were digitized at a sampling rate of 1 kHz in LabVIEW software (National Instruments, Austin, USA). During the unstable bilateral stance in the pre-test and post-test, the trajectory of the center of pressure (COP) was recorded by a force plate (Kistler Type 9260A, Switzerland). The force plate data were conditioned with an amplifier (DAQ for BioWare Type 5695B, Switzerland) and sampled at a rate of 1,000 Hz with BioWare® software (Type 2812A, Switzerland). The COP data were also synchronized with EEG measures.

### Data Analysis

The angular movement of the plate movement of the stabilometer task and COP sway in the anterio-posterior (AP) and medial-lateral (ML) directions of the transfer task were conditioned with a 4th-order low-pass Butterworth filter (cutoff frequency: 6 Hz) (Figure 1B). A set of postural sway metrics were derived from the COP sway data using established procedures: (1) root mean squared sway amplitude (unit in cm) along the AP (RMS_AP_) and ML axes (RMS_ML_); (2) 95% confidence ellipse sway area (CEA – unit in cm^2^), mean frequency (Hz), and (3) sample entropy (SampEn). Mean frequencies (MF) of the stabilometer movement and COP were determined with the power spectra of the postural data, estimated using a fast Fourier transform and the Welch method (Hanning window, window length: 15 s, overlapping time segment: 25% × window length) with a spectral resolution of 0.02 Hz (Figure 1B). The MF indexed a spectral shift in postural sway and responsiveness for postural regulation. The COP data were down-sampled to 100 Hz before sample entropy calculation. The mathematical formula of sample entropy was $SampEn\left(m,r,N\right)=-\log\left(\overset{N-m}{\underset{i=1}{\sum }}A_{i}/\overset{N-m}{\underset{i=1}{\sum }}B_{i}\right)$, where *r* = 20% of the standard deviation of the data, *m* is the length of the template (*m* = 2), and *N* is the number of data points in the time series. *A*_*i*_ is the number of matches of the *i*th template of length *m* + *1* data points, and *B*_*i*_ is the number of matches of the *i*th template of length *m* data points. Postural sway regularity is an effective biomarker to index attentional investment in postural control (Roerdink et al., 2011). In comparison to randomness, an increase in the regularity (or smaller SampEn) of sway response indicates a higher degree of attentional involvement in postural control. Task errors during the stabilometer stance (3rd−42th s) in the pre-test, in the post-test, and during the practice session (from round 1 to round 12) were indexed with the RMS value of the mismatches between the angular displacement of the stabilometer plate and the target signal. The stabilometer data of the first 2 s and the last 2 s were excluded because these data were relatively unstable. Standardized errors during the practice session were denoted as the percentage of task errors relative to the task error of the first round. The postural variables were analyzed using MATLAB R2019a software (Mathworks, USA). All of the postural variables of the three trials in the pre-test and post-test were averaged for each participant.

EEG data of the transfer task in the pre-test and post-test were analyzed. The entire EEG data were first filtered between 1 and 60 Hz using a zero-phase finite impulse response (FIR) filter (60 dB/octave) to remove the DC shift. Eye blinks were detected by creating a bipolar vertical EOG channel by subtracting activity in the infraorbitally-placed electrode from the superorbitally-placed electrode. Horizontal eye movements were detected by creating a bipolar horizontal EOG channel by subtracting activity in the electrode placed at the outer canthus of the left eye from the electrode placed at the outer canthus of the right eye. Correction of ocular artifacts was performed using regression analysis (Semlitsch et al., 1986). After eye movement was removed, EEG data of part of the run were segmented in 2 s epochs. Epochs surviving automated artifact rejection were visually inspected for undetected artifacts by researchers. All computations were performed on individual artifact-free EEG epochs. In synchronization with the COP data, no epochs were selected from the first 2 s of each run. For each subject, the weighted phase-lag index (wPLI) and variables of minimum spanning trees (MST) in the pre-test and post-test were estimated with artifact-free epochs.

The pre-processed EEG data were further filtered in the following frequency sub-bands: theta (4–8 Hz); alpha (8–13 Hz); and beta (13–20 Hz). Oscillations under 4 Hz and above 20 Hz were not analyzed because of potential contamination of muscle artifacts (van Lutterveld et al., 2017). Functional connectivity between EEG time-series of all 30 electrode pairs was calculated using the weighted phase-lag index (wPLI), an extension version of the phase-lag index (PLI). The wPLI maximizes the weight of ± 90 degree phase differences, whereas uniformly driven (such as volume conduction) sources are suppressed. The advantage of the wPLI is that it is more immune to noises than is PLI (Vinck et al., 2011) and thus recommended for analyzing cognitive dynamics within EEG during human movement (Lau et al., 2012). The traditional PLI indexes the distribution asymmetry of phase differences in the instantaneous phases of two time series, derived from the Hilbert transformation. If φ(*t*) is the phase difference, the PLI is defined as: *PLI* = |*E*{*sgn*(Δφ(*t*))}|, where *sgn* is a function that extracts the sign of a real number. Based on the PLI, the wPLI further weights each phase difference according to the magnitude of the lag:

$$\begin{array}{l} wPLI=\frac{\left|E\left\{sgn\left(\Delta \varphi \left(t\right)\right)\right\}\right|}{E\left\{sgn\left(\Delta \varphi \left(t\right)\right)\right\}}=\frac{\left|E\left\{\Delta \varphi |sgn\left(\Delta \varphi \left(t\right)\right)|\right\}\right|}{E\left\{\left|\Delta \varphi \left(t\right)\right|\right\}} \end{array}$$

The wPLI ranges between 0 (no phase synchronization) and 1 (complete phase synchronization). A low wPLI reflects continuous and uniform information flow between the electrode pairs, in comparison to a high wPLI, which implies dynamic information flow with high irregularity between the electrode pairs (Lau et al., 2012).

**Figure 2:**
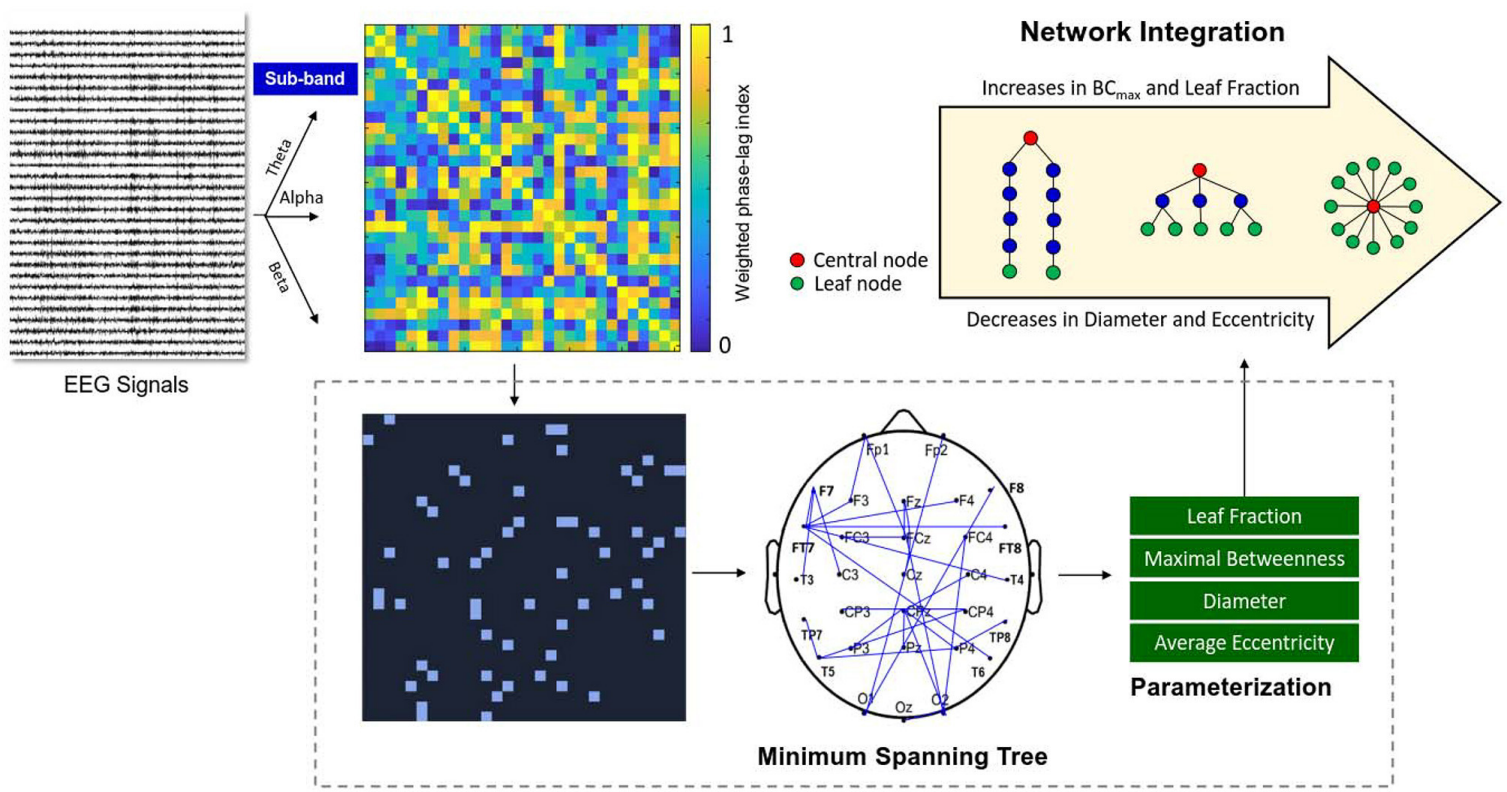
Schematic representation of network integration using variables of EEG-based minimum spanning trees (MSTs). A functional connectivity matrix of the pre-processed sub-band EEG (theta, alpha, or beta rhythms) was constructed using weighted phase-lag index (from zero to one). The MSTs consisted of the strongest connections of the functional connectivity matrix without loops. Four important variables (leaf fraction, maximal betweenness (BC_max_), diameter, and average eccentricity) were selected from the MSTs to index the degree of brain network integration.

The network topology of the wPLI functional connectivity matrix was characterized by the minimum spanning trees (MSTs) (Stam et al., 2014). Figure 2 shows a calculation pipeline of MST from artifact-free EEG epochs. Four key graph measures [diameter, leaf fraction, average eccentricity, and maximal betweenness (BC_max_)] of the MST were used to describe the network topology and the level of network integration (van Lutterveld et al., 2017). The diameter represents the length of the longest path in the network. The leaf fraction is defined as the ratio of the number of nodes with only one edge and the maximum possible number of nodes with only one edge. The BC_max_ indicates the highest value of betweenness centrality, or the number of shortest paths passing through a node, in the network. The eccentricity of a node was defined as the longest distance (the number of edges) between that node and any other node. Average eccentricity is the arithmetic mean of all nodes. In general, an integrated functional network has a higher BC_max_ and leaf fraction, together with a lower diameter and average eccentricity (van Lutterveld et al., 2017). The four MST variables were obtained and averaged from the MSTs of all artifact-free segments of the three sub-bands (theta, alpha, and beta). These MST variables of three experimental trials were averaged for each subject. Signal processing of the EEG data was performed in Matlab (Mathworks Inc. Natick, USA). The wPLI functional connectivity was calculated with the functions of HERMES for Matlab (Niso et al., 2013). The parameterization of network properties was accomplished with functions of the Brain Connectivity Toolbox (Rubinov and Sporns, 2010).

### Statistical Analysis

The primary interest of the present study was to compare performance gains and neural adaptation the transfer task after stabilometer training for the two groups. For the practice session, paired *t*-test was used to examine the difference between standardized errors of the 2nd−12th rounds and that of the first round. Paired *t*-statistics were also used to compare the CEA between the pre-test and post-test in the SV and control groups. Multi-variate Hotelling's *T*-squared statistics were used to examine the group differences (pre-test vs. post-test) in the postural sway variables of the transfer task (RMS, MF, and SampEn in the AP and ML directions) in the SV and control groups. Likewise, Hotelling's T-squared statistics were also used to examine related differences in all MST variables of the three sub-bands (theta, alpha, and beta) of the transfer task between the pre-test and post-test in the two groups. The *post-hoc* test was the Simes test, which would not produce over-correction, unlike the Bonferroni test. For all *post-hoc* hypotheses ($H=\cap_{i=1}^{m}$), the Simes test did not reject elementary *H*_*i*_ if *p*_*i*_ ≤ i^*^0.05/m for ordered unadjusted *p*-values (*p*_1_ ≤… ≤ *p*_*m*_). The type 1 error rate using the Simes test proved to be exactly 0.05. Data are presented as group means ± standard error. All statistical analyses were performed in IBM SPSS Statistics (v19). The level of significance was 0.05.

## Results

### Behavior Performance

**Figure 3:**
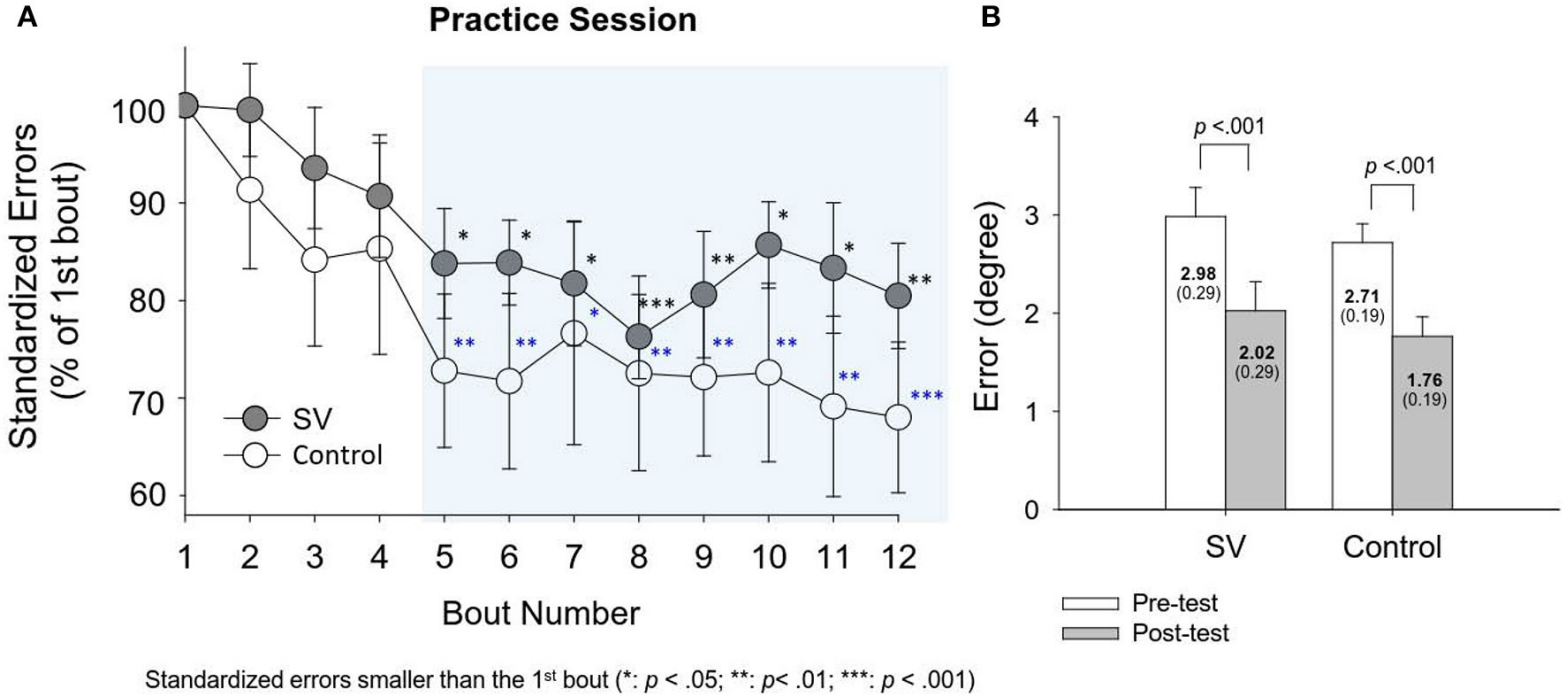
**(A)** Evolutional changes in standardized errors (% of task error of the first round) for the stabilometer task during the practice session for the SV and control groups. The standardized errors from the 5th round to the 12th round in the shaded area were significantly lower than those in the first round (*p* < 0.05). **(B)** The comparison of task errors in degrees for the trained stabilometer task between the pre-test and post-test.

The left plot of Figure 3 shows evolutional changes in standardized errors (% of the task error in the first round) between the SV and control groups during the training sessions (Day 2). In both the control and SV groups, the standardized errors decreased progressively with training sessions. The standardized errors after the fifth practice round were significantly smaller than those of the first round in both groups (*p* < 0.05). The results of paired t statistics further revealed a significant reduction in task errors of the stabilometer stance in the post-test (Day 3), as compared with that in the pre-test (Day 1) (SV: *t*_15_ = 4.805; *p* < 0.05; Control: *t*_15_ = 5.311; *p* < 0.05). Both groups exhibited significant performance gains on the trained stabilometer task.

With respect to the foam stance (the transfer task), the CEA of the post-test (3.92 ± 0.37 cm^2^) was marginally smaller than that of the pre-test (3.51 ± 0.28 cm^2^) for the control group (*t*_15_ = −2.039; *p* = 0.058). In the SV group, the CEA of the post-test (3.87 ± 0.72 cm^2^) was not different from that of the pre-test (4.08 ± 0.79 cm^2^) (*t*_15_ = 0.661; *p* = 0.519). Tables 1A,B show the results of Hotelling's T-squared statistics to compare the training-related differences in the COP variables in the AP and ML directions in the SV and control groups. In the AP direction, COP variables in the post-test differed from those in pre-test in the control (Wilks' Λ = 0.412, *p* = 0.008) and SV groups (Wilks' Λ = 0.471, *p* = 0.018) (Table 1A). In the control group, *post-hoc* analysis revealed that SampEn in the AP direction of the post-test was greater than that of the pre-test (*p* = 0.0167). The RMS of the COP trajectory in the AP direction (RMS_AP_) was smaller in the post-test than in the pre-test (*p* < 0.0333). In the SV group, RMS_AP_ was not different before and after training (*p* > 0.05). SampEn (*p* < 0.0167) and MF (*p* < 0.0333) in the AP direction increased in the post-test. In the ML direction, COP variables were insensitive to training in the control and SV groups (*p* > 0.05).

**Table 1:** The comparison of population means for center-of-pressure (COP) variables between the pre-test and post-test in the SV and control groups.

**(A) The anterioposterior (AP) direction**					
**AP characteristics**		**Pre-test**	**Post-test**	**Hotelling's statistics**	***Post-hoc*** **test**
SV	RMS (cm)	0.609 ± 0.041	0.597 ± 0.043	**Wilks' Λ = 0.412**, ***p*** **= 0.008**	*t_15_* = −0.401, *p* = 0.694
	MF (Hz)	**0.278 ± 0.018**	**0.347 ± 0.028**^††^		***t**_**15**_* **= 3.201**, ***p*** **= 0.006**
	SampEn	**0.101 ± 0.006**	**0.123 ± 0.008**^††^		***t**_**15**_* **= 4.151**, ***p*** **= 0.001**
Control	RMS (cm)	**0.650 ± 0.022**	0.603 ± 0.019^*^	**Wilks' Λ = 0.471**, ***p*** **= 0.018**	*t_15_* = −2.398, *p* = 0.030
	MF (Hz)	0.272 + 0.024	0.309 ± 0.024		*t_15_* = 1.397, *p* = 0.115
	SampEn	**0.090 ± 0.006**	**0.108 ± 0.007**^††^		***t**_**15**_* **= 3.341**, ***p*** **= 0.004**
**(B) The medio-lateral (ML) direction**					
**ML characteristics**	**Pre-test**	**Post-test**	**Hotelling's statistics**	***Post-hoc*** **test**	
SV	RMS (cm)	0.441 ± 0.050	0.463 ± 0.053	Wilks' Λ = 0.921, *p* = 0.775	
	MF (Hz)	0.251 ± 0.020	0.267 ± 0.019		
	SampEn	0.085 ± 0.006	0.081 ± 0.007		
Control	RMS (cm)	0.443 ± 0.037	0.430 ± 0.027	Wilks' Λ = 0.768, *p* = 0.314	
	MF (Hz)	0.240 ± 0.012	0.200 ± 0.010		
	SampEn	0.070 ± 0.004	0.069 ± 0.003		

†† *Post-test > Pre-test, p < 0.0167*.

* *Post-test < Pre-test, p < 0.0333*.

*RMS: root mean square; MF: mean frequency; SampEn: sample entropy*.

*Bold values highlight the statistic results with significant difference*.

### EEG MST Analysis

Tables 2A–D summarize training-related changes in network graph measures of the transfer task (unstable bilateral stance) in the SV and control groups. In the control group, the results of Hotelling's T-squared statistics revealed a significant difference in MST diameter between the pre-test and post-test (Wilks' Λ = 0.395, *p* = 0.006) (Table 2A). The diameters of the alpha band (*p* < 0.0167) and beta band (*p* < 0.0333) in the post-test were significantly smaller than those in the pre-test. In contrast, the MST diameter did not differ between the pre-test and post-test in the SV group (*p* > 0.05). In the control group, there was a significant difference in the leaf fraction between the pre-test and post-test (Wilks' Λ = 0.430, *p* = 0.010) (Table 2B). The leaf fraction of the beta band (*p* < 0.0167) in the post-test was larger than in the pre-test, and the post-test leaf fraction of alpha band was marginally increased (*p* = 0.047). In contrast, the leaf fraction of the SV group did not vary with training (*p* > 0.05). The average eccentricity between the pre-test and post-test was training-dependent (Wilks' Λ = 0.535, *p* = 0.038) in the control group rather than in the SV group (*p* > 0.05) (Table 2C). In the control group, the average eccentricity of the alpha band was significantly smaller in the post-test than in the pre-test (*p* < 0.0333), while the average eccentricity of the beta band was marginally smaller in the post-test (*p* = 0.027). Likewise, only the control group exhibited training-related mediation of BC_max_ (Wilks' Λ = 0.453, *p* = 0.014); the SV group did not (*p* > 0.05). In brief, after stabilometer training, the SV group did not significantly mediate MST measures for the transfer task like the control group did.

**Table 2:** The comparison of population means for network integration graph measures between the pre-test and post-test in the SV and control groups.

**(A) Diameter**						
**Diameter**		**Pre-test**	**Post-test**	**Hotelling's statistics**	***Post-hoc*** **test**
SV	Theta	0.347 ± 0.009	0.356 ± 0.006	Wilks' Λ = 0.719, *p* = 0.217	
	Alpha	0.381 ± 0.012	0.353 ± 0.011		
	Beta	0.357 ± 0.007	0.341± 0.008		
Control	Theta	0.350 ± 0.009	0.335 ± 0.011	**Wilks' Λ = 0.395**, ***p*** **= 0.006**	*t_15_* = −1.442, *p* = 0.170
	Alpha	**0.371 ± 0.009**	0.332 ± 0.009^**^		***t**_**15**_* **= −3.164**, ***p*** **= 0.006**
	Beta	**0.372 ± 0.007**	0.346 ± 0.008^*^		***t**_**15**_* ** = −2.584**, ***p*** **= 0.021**
**(B) Leaf fraction**					
**Leaf Fraction**		**Pre-test**	**Post-test**	**Hotelling's statistics**	***Post-hoc*** **test**
SV	Theta	0.539 ± 0.010	0.538 ± 0.009	Wilks' Λ = 0.963, *p* = 0.168	
	Alpha	0.517 ± 0.012	0.527 ± 0.010		
	Beta	0.543 ± 0.015	0.545 ± 0.010		
Control	Theta	0.536 ± 0.014	0.552 ± 0.010	**Wilks' Λ = 0.430**, ***p*** **= 0.010**	*t_15_* = 1.095, *p* = 0.291
	Alpha	0.510 ± 0.012	0.527 ± 0.010		*t_15_* = 2.162, *p* = 0.047
	Beta	**0.530 ± 0.010**	**0.566 ± 0.011^††^**		***t**_**15**_* **= 2.758**, ***p*** **= 0.015**
**(C) Eccentricity**					
**Average Eccentricity**		**Pre-test**	**Post-test**	**Hotelling's Statistics**	***Post-hoc*** **test**
SV	Theta	0.275 ± 0.007	0.282 ± 0.005	Wilks' Λ = 0.755, *p* = 0.286	
	Alpha	0.295 ± 0.008	0.279 ± 0.008		
	Beta	0.280 ± 0.009	0.270 ± 0.006		
Control	Theta	0.276 ± 0.008	0.265 ± 0.008	**Wilks' Λ = 0.535**, ***p*** **= 0.038**	*t_15_* = −1.385, *p* = 0.186
	Alpha	**0.294 ± 0.008**	0.269 ± 0.008^*^		***t**_**15**_* ** = −2.425**, ***p*** **= 0.028**
	Beta	0.293 ± 0.006	0.274 ± 0.006		*t_15_* = −2.454, *p* = 0.027
**(D) Maximal betweenness (BC**_**max**_**)**					
**BC**_**max**_		**Pre-test**	**Post-test**	**Hotelling's statistics**	***Post-hoc*** **test**
SV	Theta	0.703 ± 0.013	0.717 ± 0.014	Wilks' Λ = 0.877, *p* = 0.621	
	Alpha	0.703 ± 0.013	0.704 ± 0.010		
	Beta	0.702 ± 0.010	0.719 ± 0.013		
Control	Theta	0.709 ± 0.009	0.729 ± 0.014	**Wilks' Λ = 0.453**, ***p*** **= 0.014**	*t_15_* = 1.750, *p* = 0.101
	Alpha	0.697 ± 0.006	0.726 ± 0.009		*t_15_* = 2.655, *p* = 0.018
	Beta	0.704 ± 0.009	0.719 ± 0.012		*t_15_* = 1.115, *p* = 0.282

* *Post-test < Pre-test, p < 0.0333*.

** *Post-test < Pre-test, p < 0.0167*.

†† *Post-test > Pre-test, p < 0.0167*.

*Bold values highlight the statistic results with significant difference*.

## Discussion

This study revealed a significant visual impact on postural skill transferability in older adults. Despite a comparable training benefit on stabilometer stance for the control and SV groups, the transfer effect on sway reduction in foam stance (particularly COP RMS in the AP direction) was confined to the participants trained under the full-vision feedback. Contrary to the control group, the SV group who trained under the intermittent vision feedback did not show significant integration of supraspinal neural network to execute the posture transfer task.

### Comparable Training Specificity With Stroboscopic Vision and Full Vision

A stabilometer, which addresses foot-support interaction, is commonly used to train the dynamic standing-balance ability in rehabilitation clinics. After stabilometer training, the SV and control groups consistently showed training benefits, with a significant reduction in size of postural sway during the stabilometer stance (Figure 3, right). However, intermittent visual feedback did not produce additional performance gain on stabilometer stance, probably because stroboscopic vision could also add to the effectiveness of using residual visual input and/or other sensory facilities, such as the proprioceptive and vestibular systems (Wilkins and Appelbaum, 2019). In addition, stroboscopic vision is expected to reinforce compensatory recruitment of attentional brain resources [such as frontal/prefrontal networks (Huang et al., 2016, 2017)] for visual-motor processing associated with a specific postural task. However, stroboscopic vision also increased task difficulty, which accompanies by higher contextual interference on the stabilometer stance. According to the frontal aging hypothesis (Lalonde and Badescu, 1995; D'Esposito and Chen, 2006), additional task load could tax limited frontal attentional resource of the older adults that counterbalanced potential training benefits of compensatory recruitment of the brain with stroboscopic vision. On the other hand, the comparable training benefits on stabilometer stance allowed us to largely rule out the possibility that the learning phase performance affects the transfer performance. In fact, the transferability of postural skill from the stabilometer stance to the unstable bilateral stance was only evident in the control group, granting that COP RMS in the AP direction during unstable bilateral stance was reduced with stabilometer training for the control group rather than for the SV group (Table 1A). The directionally dependent training benefits in the AP direction can be explained by training specificity, because stabilometer training mainly provides a scalable balance constraint on controls of the ankle joint in plantarflexion and dorsiflexion (Donker et al., 2007). In line with the idea of training specificity, both the SV and control groups exhibited training-related increase in the SampEn of COP sway in the AP direction, in support of a higher degree of automated postural control in the post-test. Together with the lack of training-related decline in the size of COP sway, the SV group exhibited a training-related higher mean frequency of COP sway in the AP direction during the posture transfer task (Table 1A). The scenario reflected stance uncertainty on the unstable foam stance with increasing demand on sensory feedback and/or postural correction attempts (Davis et al., 2009; Goh et al., 2016).

### The Lack of Postural Skill Transfer With Stroboscopic Vision Training

Intriguingly, motor transfer to the foam stance was limited with the use of stroboscopic vision in the older adults. Hence, mastery of a motor skill differs from motor transfer, which depends on how the original motor memory is coded in the associative phase of motor learning (Censor, 2013; Wang and Song, 2017; Herszage and Censor, 2018). Degenerative changes in the hippocampus and associated medial temporal structures can cause generalization deficits in older adults (Krishna et al., 2012; Simon and Gluck, 2013). Hippocampal damage disrupts learned association for a lack of context-specific stimulus representation (Myers et al., 2002). Therefore, contextual visual feedback plays an important role in the relational binding of motor memories to generalize invariant features in space and time (Turk-Browne, 2019), considering the rich hippocampus-vision interconnection and the ineffectiveness of postural skill transfer under training with stroboscopic vision. Also, intermittent visual feedback, which decreases the certainty about the predicted success of a postural goal, is disadvantageous to the construction of an associative network to generalize prior stabilometer learning. Our results were in agreement with a recent study (Shalmoni and Kalron, 2020), which trained multiple sclerosis patients under conditions of intermittent vision for ball-catching tasks. The authors also found no training-related differences in pre-post measures for gait and balance following the stroboscopic training. Therefore, adequate visual feedback is keyed to postural skill transfer in older adults, who already have an increased reliance on the visual system for postural control. The behavioral results highlight the fact that visual cues can improve plan-based posture control in older adults when explicit awareness of posture-relevant features is desirable under environmental constraints (Caljouw et al., 2016).

With an MST approach, this study first revealed increased network integration for the transfer postural task with full-vision feedback relative to that of the intermittent visual feedback. Motor transfer is a top-down process, involving hippocampal-cortical activity to modulate similarity-based memory coding (Kahnt and Tobler, 2016) and memory reactivation of past experiences (Yu et al., 2018). Central to motor transfer, the hippocampus is connected to the cortical regions, including prefrontal/frontal cortex for error monitoring/attentional gating (Crochet et al., 2019), the medial cingulate cortex for encoding and retrieval of associative memory (Caviezel et al., 2020), the medial temporal lobe for memory storage/consolidation, the visual cortex for association of visuomotor execution (Seidler, 2010; Turk-Browne, 2019), and so on. These hippocampal–cortical activities suggest that variations in the hub nodes and information flow of the MST are concentrated in the cortical areas at generalization. Functional connectivity tends to have a star-shaped topology for the execution of a transfer task of unstable bilateral stance, in support of the increased leaf fraction (Table 2B). In addition, an increase in BC_max_ (Table 2D) indicates that greater key routes of information flow converge at one or a few hub nodes for the transfer task. Negatively correlated to network integration, decreases in the average eccentricity and MST diameter indicate higher network efficiency for transfer task completion (Tables 2A,C). After stabilometer training, the control group could exploit a more centrally-organized network with increasingly closer nodes to the central node during the transfer task. With oscillatory mechanisms to facilitate anticipation and processing of visual information (Kilavik et al., 2013; Jensen et al., 2015), the increase in alpha/beta functional networks for the control group was likely to reinforce large-scale communication between the sensorimotor and other areas and the periphery for the transfer task. In a relatively stable upright stance, a smaller sensorimotor conflict could enhance alpha–beta synchronization in the occipito-parietal areas, and vice versa (Peterson and Ferris, 2018). In contrast, the above-mentioned network reorganization was almost lacking in the SV group trained with intermittent vision (Tables 2A–D).

## Conclusion

For older adults, stroboscopic vision results in comparable training benefits for postural straining on stabilometer as full-vision. Contrary to the expectation, compared with full-vision feedback, postural skill transfer from stabilometer training to foam stance was impaired with intermittent vision. Postural training with stroboscopic vision fails to enhance integration of supraspinal neural network for executing the postural transfer task. The behavioral and neural scenarios agree that full vision is necessary to code transferable postural skills for the older adults.

## Data Availability Statement

The raw data supporting the conclusions of this article will be made available by the authors, without undue reservation.

## Ethics Statement

The studies involving human participants were reviewed and approved by Institutional Review Board (IRB) at the National Cheng Kung University (NCKU) Hospital, Taiwan. The patients/participants provided their written informed consent to participate in this study.

## Author Contributions

I-SH: conception or design of the work and final approval of the version to be published. Y-CCho: acquisition. Y-CChe and I-SH: analysis, interpretation of data, and drafting the work or revising it critically for important intellectual content. Y-CChe, Y-CCho, and I-SH: agreement to be accountable for all aspects of the work in ensuring that questions related to the accuracy or integrity of any part of the work are appropriately investigated and resolved. All authors contributed to the article and approved the submitted version.

## Conflict of Interest

The authors declare that the research was conducted in the absence of any commercial or financial relationships that could be construed as a potential conflict of interest.

## Abbreviations

AP: anterio-posterior
BC_max_: the highest value of betweenness centrality
CEA: confidence ellipse sway area
COP: center of pressure
EEG: electroencephalography
EOG: electrooculography
FIR: finite impulse response filter
ML: medial-lateral
MF: mean frequency
MST: minimum spanning tree
PLI: phase-lag index
RMS_AP_: root mean squared sway amplitude along the anterio-posterior direction
RMS_ML_: root mean squared sway amplitude along the medial-lateral direction
SampEn: sample entropy
SV: stroboscopic vision
wPLI: weighted phase-lag index.
